# Mesenteric panniculitis in a child misdiagnosed as appendicular mass: a case report and review of literature

**DOI:** 10.1186/2193-1801-3-73

**Published:** 2014-02-06

**Authors:** Nisreen Rumman, George Rumman, Barakat Sharabati, Rami Zagha, Nimer Disi

**Affiliations:** Department of Pediatrics, Makassed Hospital, Jerusalem, Palestine; Department of Pediatric Surgery, Makassed Hospital, Jerusalem, Palestine; Department of Pathology, Makassed Hospital, Jerusalem, Palestine; Department of Pathology, Al-Najah National University, Nablus, Palestine

## Abstract

Mesenteric panniculitis is a chronic inflammatory process involving the adipose tissue of the mesentery. The etiology is unknown, and it is rare in children. We report a 5 year old girl who presented with abdominal symptoms and was misdiagnosed as appendicular mass. The correct diagnosis was established after surgical resection.

## Introduction

Mesenteric panniculitis is a rare disease characterized by chronic inflammation of the adipose tissue of the mesentery. The specific etiology is unknown, but surgery, trauma, infection, autoimmunity and malignancy have been suggested as possible factors.

Most of the cases described in the literature are adults. The disease is exceptional in children probably because of less mesenteric fat. We report here a case of mesenteric panniculitis in a child who presented with abdominal pain following a minor trauma, and was misdiagnosed initially as appendicular mass.

### Case description

A five year old female child was referred for evaluation of an abdominal mass. She has had a history of minor abdominal trauma one month prior when she fell down while playing. Her abdominal ultrasound at that time showed minimal intraabdominal free fluid. She was observed for one day at another hospital and discharged home the following day without any symptoms. Ten days later she started to have severe right sided abdominal pain, associated with fever (up to 38.5°C) and poor oral intake, but no vomiting or diarrhea. She had leukocytosis (WBCs 18,000 N 69%), and abdominal ultrasound showed a non specific abdominal mass on the right side. A laparotomy was done for suspicion of an appendicular mass. The mass was not excised but a biopsy and culture were taken and a drain left in place. Her fever and abdominal pain persisted postoperatively with new onset of vomiting.

**Figure 1:**
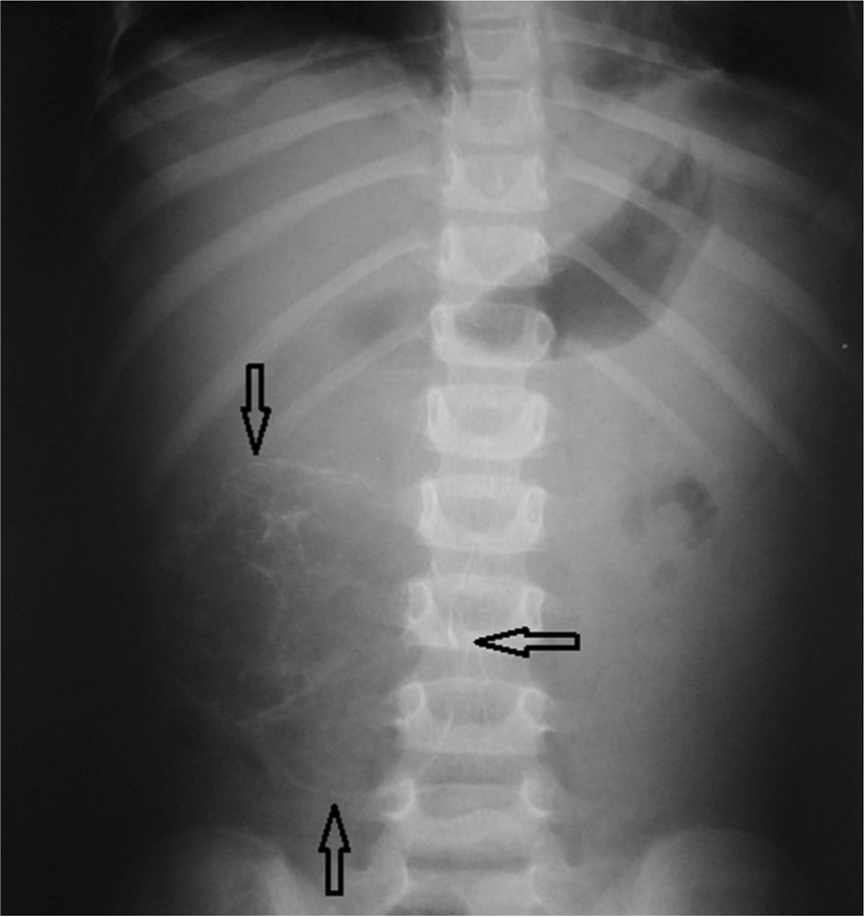
**Abdominal x-ray showing a large mass in the right side occupying the whole lumbar region.** The mass is heterogeneous in consistency with rims of calcifications. The arrows point to the borders of the mass.

**Figure 2:**
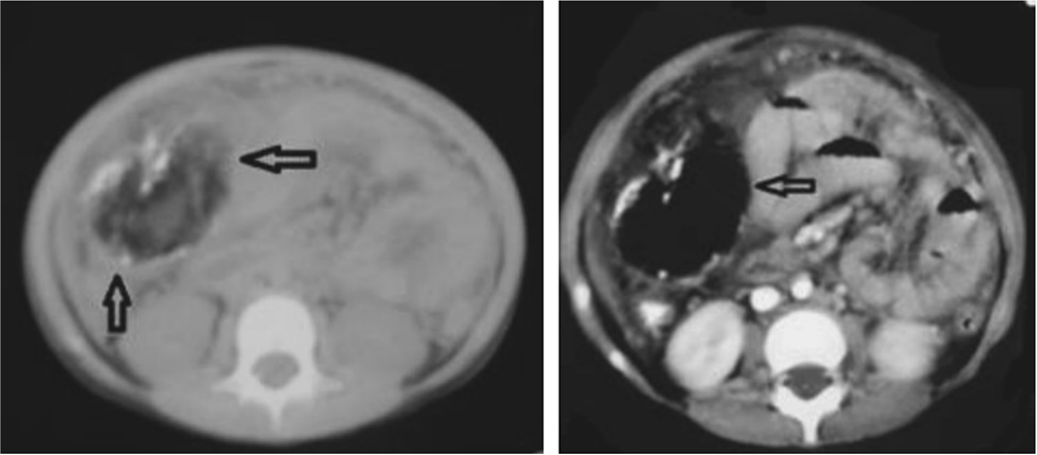
**CT images of the abdomen showing the large right sided mass with inflammatory changes and calcifications within the fatty tissues of the mesentery.**

**Figure 3:**
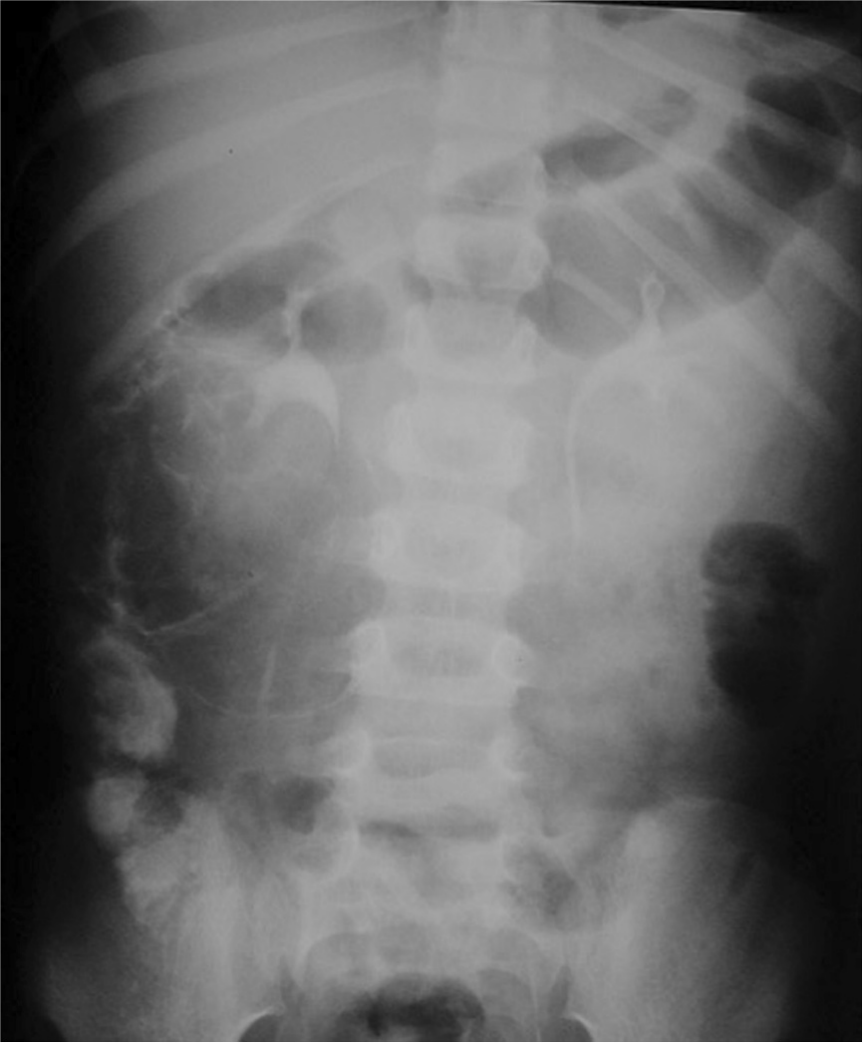
**Intravenous pyelography showing normal right pelvicaliceal system with no dilatation, compression or obstruction to the flow of contrast.** There is no relation to the intra-abdominal mass.

**Figure 4:**
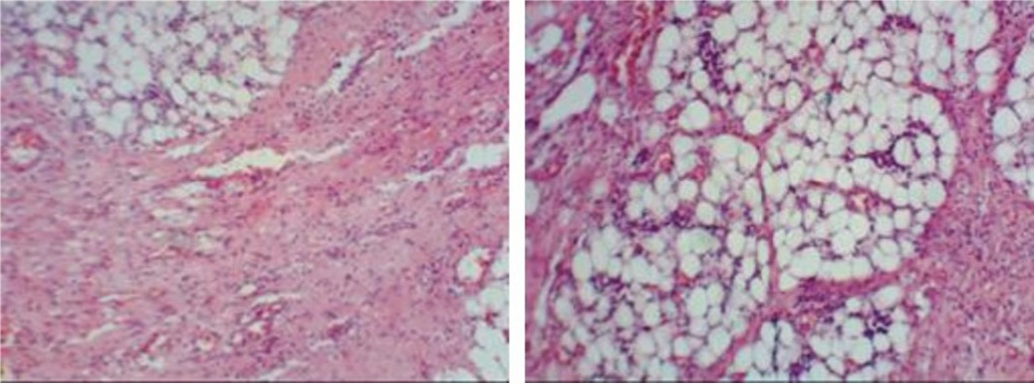
**Pathology slides of the mass showing areas of necrosis, mixed inflammatory infiltrates and fibroblast proliferation within adipose tissue consistent with mesenteric panniculitis.**

On arrival to our hospital she was still in pain and febrile (38.5-39°C). She was irritable with right sided abdominal tenderness. Her labs showed leukocytosis with neutrophilia (WBCs 14.600, N 76.2%, L 17.3%), ESR 38, kidney and liver function tests were within normal. Abdominal X-ray showed a right sided mass with calcifications (Figure 1). Abdominal CT scan confirmed an intraabdominal mass on the right side below the transverse colon, with calcified walls and characteristics compatible with inflammatory fatty tissue (Figure 2). Intravenous pyelography revealed no relation to the right kidney (Figure 3). She was initially managed conservatively with IV fluids, antibiotics and bowel rest. She improved clinically, fever and pain subsided and inflammatory markers decreased. She was discharged home with close follow up of the mass. Twenty days later, the mass had increased in size, so she underwent an exploratory laparotomy. The intraperitoneal (10 × 7 cm) mass was found to be hypervascularized with significant adhesions to the surrounding bowel and the omentum covering it. The mass was resected without complications. The patient improved and all her symptoms resolved. Pathology showed fibroadipose tissue displaying areas of adiponecrosis, foci of mixed inflammatory infiltrate, and proliferation of bundle forming fibroblasts consistent with mesenteric panniculitis (Figure 4).

## Discussion

Mesenteric panniculitis (MP) is a chronic fibrosing inflammation of the mesentery of the small intestines and colon (Issa & Baydoun 2009). It is generally rare although some believe it is under-diagnosed and under-reported (Nicholson et al.). Interestingly, a review by Nicholson et al. noted that none of the 10 consultant surgeons and only one of the twelve radiologists at their institution had any knowledge of this condition. It has been estimated to affect 1% of the population (Nicholson et al.).The incidence increases with age being more frequent in the fifth to seventh decades of life (Ferrari et al. 2008; Akram et al. 2007). It is more common in Caucasian men with a male to female ratio of 2–3: 1. It is thought to be less frequent in children perhaps because of less amount of mesenteric fat compared to adults (Issa & Baydoun 2009; Delgado Plasencia et al. 2007).

MP remains a poorly understood phenomenon and the exact etiology is still unknown. Many predisposing factors have been described. The most commonly reported associations were surgery and abdominal trauma (Issa & Baydoun 2009; Emory et al. 1997) with the assumption that MP may represent an abnormal healing process to tissue injury (Wilkes et al.). As an example, the exposure to continuous vibration of a pneumatic jackhammer was thought to be the cause of MP in a construction worker (van der Hulst et al. 1995). MP followed colonoscopy, polypectomy and epinephrine injection that was used to control bleeding in another patient (Lee et al.). Other theories postulated ischemia and infection such as tuberculosis as causative factors (Ege et al. 2002). A strong association with tobacco use has been reported as well (Delgado Plasencia et al. 2007).

Wilkes et al. hypothesized that MP may represent a paraneoplastic phenomenon. They identified malignancy in 45 out of 118 patients with MP. The most common were colorectal, urogenital malignancies and lymphoma. They even identified some factors as predictors of subsequent development of malignancy in MP patients; a lymph node size >12 mm and absence of the fat ring sign (preservation of fat immediately adjacent to superior mesenteric vessels) (Wilkes et al.). Daskalogiannaki et al. also found that CT findings of MP coexisted with malignancy in 69.4% of cases (Daskalogiannaki et al. 2000). A wide variety of systemic diseases have been associated with MP like vasculitis (Weber-Christian disease), rheumatological diseases like systemic lupus erythematosus and Henoch-Schonlein purpura (Martin-Sune et al.), granulomatous disease and pancreatitis (Ege et al. 2002). Association with celiac disease (Sampert et al.) and infiltration with cells expressing IgG4 (Akram et al. 2007; Belghiti et al. 2009) suggest possible autoimmune process.

The main pathological changes include a spectrum of chronic, non-specific inflammation, fat necrosis and fibrosis. The disease is known in the literature by many other names based on the predominant pathologic component. MP is the term used when inflammatory changes are predominant. Mesenteric lipodystrophy is used when there is predominant adipose tissue necrosis. Retractile or sclerosing mesenteritis is characterized by fibrosis that thickens and shortens the mesentery hence the name retractile (Piessen et al. 2006). The latter is considered the final more invasive stage (Daskalogiannaki et al. 2000). Many other names are referred to in the literature reflecting the histological variation across cases: mesenteric liposclerosis, mesenteric manifestations of Weber-Christian disease, xanthogranulomatous mesenteritis, inflammatory pseudotumor, mesenteric lipogranuloma, mesenteric lipomatosis, and systemic nodular panniculitis (Delgado Plasencia et al. 2007). There is no consensus on nomenclature, and it is uncertain whether these represent separate entities or merely different presentations of the same underlying process (Cheng & Liu 2008). Emroy et al. suggested that sclerosing mesenteritis would be the most appropriate term (Emory et al. 1997).

MP is most commonly reported in the mesentery of small intestine (90% of cases) (Delgado Plasencia et al. 2007) especially the jejunum (Wilkes et al.). But it can involve the colonic mesentery (Oomori 2007), the sigmoid (Popkharitov & Chomov 2007), the peripancreatic and omental fat (Delgado Plasencia et al. 2007; Huang et al.). Sclerosing mesenteritis can involve the parenchyma of the pancreas and mimic pancreatic cancer (Scudiere et al.).

The presentation of the disease varies considerably. It could be asymptomatic in 30-50% of cases (Ferrari et al. 2008; Piessen et al. 2006), or it may present with a wide array of symptoms. In a review by Akram et al. (Akram et al. 2007), the most frequent presenting symptoms were abdominal pain, bloating, distention, and diarrhea. Less frequent symptoms include nausea, vomiting, weight loss and constipation. Rare presentations include rectal bleeding, jaundice, gastric outlet obstruction, and acute abdomen (Delgado Plasencia et al. 2007). Fever of unknown origin has also been reported (Ferrari et al. 2008; Hemaidan et al. 1999; Hirano et al.). Pedal edema could occur secondary to mechanical effects on the bowel and vascular structures (Zafar et al. 2008).

MP usually has a benign slow course. Nevertheless, significant morbidity and mortality have been related to MP including small bowel obstruction, perforation and pneumoperitoneum (Chawla et al. 2009), ischemic colitis (Daumas et al.; Amor et al.), chylous ascites and vascular thrombosis (Akram et al. 2007). Recurrent pleural effusions occurred in a patient with severe MP, who 3 years later, was diagnosed with malignant mesothelioma (Harris et al. 1994). Biliary and pancreatic fistulae were also reported in a case of omental panniuclitis by hepatocellular carcinoma (Shindo et al.). Several fatal cases of sclerosing mesenteritis have been reported suggesting that it could be a serious disease (Andersen et al. 1982; Soergel & Hensley 1966; Kida et al.).

Because of this wide variety of non-specific presentations, MP should be in the differential diagnosis of chronic abdominal pain especially when more common causes have been excluded. Physical examination is also non-specific, abdominal tenderness and feeling of an ill defined mass may be the only findings. Signs of intestinal obstruction may also be present (Delgado Plasencia et al. 2007).

MP is rare in children. Viswanathan et al. reported a 6 year old girl with idiopathic sclerosing mesenteritis (Viswanathan & Murray). They also reviewed another 16 published pediatric cases. In this pediatric review of total 17 cases, they found no gender predominance. The age at diagnosis ranged from 18 months to 12 years with an average of 6.5 years (Viswanathan & Murray).

Similar to adults, the most common presenting feature in children was abdominal pain. Some presented with features of acute abdomen and required surgery (Cakmak et al. 1986; Duman et al.; Hakguder et al. 2000).

The differential diagnosis of MP is broad. It could easily be missed as mesenteric neoplasm (Jain et al.) or any other disorder affecting the mesentery. The most commonly encountered are lymphoma, carcinoid tumor, lipoma, liposarcoma, desmoid tumor, and retroperitoneal fibrosis (Ferrari et al. 2008; Delgado Plasencia et al. 2007).

Blood tests are usually unremarkable. Non specific elevation of inflammatory markers is common (Issa & Baydoun 2009; Nicholson et al.; Ferrari et al. 2008; Delgado Plasencia et al. 2007). Anemia related to chronic inflammation (Issa & Baydoun 2009; Delgado Plasencia et al. 2007) and hypoalbuminemia were seen in few cases (Ferrari et al. 2008; Hillemand et al.).

CT scan has been proposed as the imaging modality of choice. Some suggested that changes are classical that the diagnosis can be made by CT alone (Amor et al.). The appearance on CT can range from subtle increased attenuation in the mesentery to a solid soft tissue mass (Horton et al. 2003), but two characteristic features are considered specific: the fat ring sign and the tumoral pseudocapsule (Ferrari et al. 2008; Delgado Plasencia et al. 2007; Horton et al. 2003). Cystic components have also been described. Calcifications may be present and may be related to fat necrosis (Akram et al. 2007; Horton et al. 2003). In our patient there was a rim of calcifications within the mass evident on both plain film (Figure 1) and CT scan (Figure 2). MP can be an incidental finding on CT scan as well (Akram et al. 2007). In the radiology series by Daskalogiannaki, 7620 abdominal CT scans were reviewed and the prevalence was found to be 0.6% (Daskalogiannaki et al. 2000). Coulier et al. reported that positron emission tomograpgy (PET) scan has proved useful to exclude mesenteric tumoral involvement (Coulier). Plain abdominal X-ray and barium studies have not been shown very helpful in diagnosis. Ultrasound is a helpful non invasive imaging study with good correlation of the sonographic features of MP with those of CT findings (Roson et al. 2006). There is little data about the utility of MRI in the diagnosis of MP. Endoscopic procedures are usually unrevealing since the pathology is extrinsic to the bowel. There was only one report of the endoscopic features of MP by double ballon enteroscopy showing duodenal varices, villous hypertrophy and vascularity, inflammatory distal ileal stricturing with rectal varices (Bourikas et al.). The definitive diagnosis is confirmed by surgical biopsy which remains necessary to exclude underlying infection or malignancy (Ferrari et al. 2008; Delgado Plasencia et al. 2007; Piessen et al. 2006; Horton et al. 2003; Ehrenpreis et al. 2009; Gu et al. 2008). In some cases the diagnosis is only made after surgical resection.

MP has been reported to regress spontaneously in most patients (Nicholson et al.; Delgado Plasencia et al. 2007) and may not require any treatment. In some symptomatic cases, the response to steroids was excellent and resulted in complete resolution of the mass (Issa & Baydoun 2009; Ferrari et al. 2008). There is no consensus or specific guidelines regarding the best treatment regimen. Immunosuppressive agents like azathioprine (Tytgat et al. 1980), cyclophospahmide (Bush et al. 1986), methotrexate (Sampert et al.) have been used. Other proposed therapies include colchicine (Genereau et al. 1996; Fasoulas et al.), tamoxifen (Venkataramani et al. 1997), oral progesterone (Mazure et al. 1998) and thalidomide (Ginsburg & Ehrenpreis 2002). Percutaneous drainage of the fluid component in MP if liquefaction is evident on CT scan can help to reduce its size (Wen & Chen 2009). Infliximab was used in a child with sclerosing mesenteritis and celiac disease (Sampert et al.).

Surgical resection is generally not recommended except in presence of complications like intestinal obstruction and ischemia (Ferrari et al. 2008; Piessen et al. 2006). In our case, the biopsy that was taken at the other hospital was inconclusive and the diagnosis was made only after complete surgical resection; therefore, medical treatment with steroids or other agents was not attempted. The mass was increasing in size which was worrisome and hastened our decision for resection. Several other cases in the literature were only diagnosed after surgical resection (Duman et al.).

The prognosis is good with a benign course and favorable outcome in most cases (Ferrari et al. 2008; Emory et al. 1997; Piessen et al. 2006). However, knowledge of this entity is essential for early correct diagnosis and avoidance of unnecessary aggressive interventions.

## Conclusion

MP is a benign condition with no definitive causal relationships, though many conditions as surgery, trauma, malignancy, autoimmunity and infection have been proposed as predisposing factors. It has various presentations with a wide range of clinical and radiological manifestations, posing a diagnostic challenge. It is important especially for the surgeons to keep this entity in the differential diagnosis of chronic ill defined abdominal pain and/or mass. Very few cases have been reported in children. Our patient was initially misdiagnosed as appendicular mass. Indeed the accurate diagnosis was difficult before surgery given the rarity of the condition in children and the lack of awareness among surgeons and radiologists about this disease. MP could be more common than we think, but rather under-diagnosed. It is possible with the wide availability of CT scans and their use in evaluating chronic ill defined abdominal pain in all age groups that more and more cases will be reported in children.

## Patient consent

Written informed consent was obtained from the patient’s parents for the publication of this report and accompanying images.
